# Speech versus noise in pediatric sound localization

**DOI:** 10.1007/s00405-026-10415-5

**Published:** 2026-07-19

**Authors:** Elisabeth Zangerl, Franz Muigg, Josef Seebacher, Simone Graf, Andre Morsnowski, Katharina Schmidt, Philipp Zelger

**Affiliations:** 1 Medical University of Innsbruck: Medizinische Universitat Innsbruck, Innsbruck, Austria; 2 University Hospital for Hearing, Speech & Voice Disorders, Tirol Kliniken Innsbruck, Innsbruck, Austria; 3 Department of Otorhinolaryngology, Head and Neck Surgery, Hospitals of the City of Cologne, Cologne, Germany; 4 Department of Otorhinolaryngology, Proselis GmbH, Prosper-Hospital, Recklinghausen, Germany; 5 Institut für Hörtechnik und Audiologie, Jade Hochschule, Oldenburg, Germany

**Keywords:** Audiology, Directional hearing, Pediatric audiology

## Abstract

**Background:**

Accurate sound localization is essential for children’s communication, safety, and learning. While noise stimuli are commonly used in directional hearing tests in clinical settings, speech stimuli may offer more child-friendly assessments.

**Objectives:**

This study aimed to compare sound localization performance using noise and word stimuli across several groups of children with different types of hearing.

**Methods:**

A total of 108 children participated in the study: 31 normal-hearing children aged 6–10 (**Ref**_**6–10**_), 32 children aged 6–10 with hearing aids (**HA**_**6–10**_), and 45 children aged 11–17 with hearing aids (**HA**_**11–17**_). Participants completed a sound localization task using five speakers placed at horizontal angles (± 90°, ± 45°, 0°). They localized broadband noise bursts or one of four German words, presented randomly at different sound levels. Localization accuracy was measured using root mean square error (RMSE). Linear mixed-effects models analysis was conducted with Stimulus and Angle as within-subject factors and Group as a between-subject factor.

**Results:**

No significant differences have been found in the comparison between stimuli regarding the RMSE between the groups. A weakly significant difference between stimulus types was found only in the HA_6–10_ group at 0°, where noise stimuli were localized more accurately than words (*p* = 0.03).

**Conclusions:**

Both noise and word stimuli are suitable for assessing directional hearing in children. However, noise stimuli are more economical, as they require fewer presentations. In contrast, word stimuli are more time and resource-demanding due to the need for multiple presentations of each word to obtain statistically reliable results.

**Supplementary Information:**

The online version contains supplementary material available at https://doi.org/10.1007/s00405-026-10415-5.

## Introduction

Sound localization, or the ability to determine the direction of a sound source, is a critical auditory skill that supports communication, learning, and safety in everyday life. For children, accurate localization helps them identify who is speaking in a classroom or detect approaching vehicles in noisy environments [1]. In normal-hearing children, this ability evolves with age. Even toddlers can perform simple sound localization tasks, but their performance typically shows larger errors compared to adults. Three-year-olds tend to localize sounds less accurately than four- or five-year-olds, reflecting the ongoing maturation of auditory spatial processing during early childhood [2–4].

In contrast, children with hearing impairment often experience significant deficits in localization. Hearing loss disrupts the binaural cues, such as interaural time and level differences, that underpin accurate directional hearing. As a result, children with unilateral hearing loss or those using hearing devices, such as hearing aids (HAs) or cochlear implants (CIs), typically exhibit reduced spatial hearing abilities compared to their normal-hearing peers [5–7]. This impairment can negatively affect situational awareness, speech understanding in noise, and overall auditory development, highlighting the importance of accurately assessing localization performance in both normal-hearing and hearing-impaired children [8, 9].

A variety of acoustic stimuli have been used in sound localization research, particularly with pediatric populations. A large number of studies employ transient broadband noise bursts (e.g., clicks or pink/white noise), as these signals provide sharp onsets and a wide spectral range, which support robust localization through interaural cues [10]. In contrast, speech-based stimuli, such as familiar words, have been used less frequently. One notable example is the use of the spondaic word “baseball” in pediatric localization testing, which was found to improve children’s engagement and task performance compared to noise bursts [8, 11, 12]. Such studies suggest that word stimuli may be more suitable or motivating for young listeners, even though they are acoustically more complex.

Noise and word stimuli differ in their acoustic properties in ways that may influence localization performance. Noise bursts typically have a single, sharp onset and broad frequency spectrum, offering clear temporal and level cues for spatial hearing. In contrast, words unfold over time and include multiple syllables and softer onsets, with energy concentrated in mid-frequency bands [1]. Prior studies in adults have shown that speech stimuli are generally localized less accurately than broadband noise, likely due to their reduced spectral sharpness and less abrupt acoustic profile [13–15].

The distinct onset makes it easier for the auditory system to detect which ear received the sound first, enhancing interaural timing difference (ITD) cues for direction [16, 17]. In contrast, gradual or multiple-onset sounds (for example, a slowly rising tone or a complex stimulus with several amplitude bursts) spread their energy over time. A slow rise-time sound may not trigger the same immediate orientation reflex in a child, potentially leading to less accurate or delayed localization since there isn’t a sudden cue to “grab” the auditory system’s attention. Research suggests that when onset cues are diminished or extended (such as using a long fade-in), localization becomes more challenging, even for adults. Children, whose spatial hearing is still developing, can be especially affected by the lack of a salient onset. Moreover, when multiple onsets occur in quick succession (such as a direct sound followed shortly by an echo or another sound), the auditory system must decide which onset to use for localization. Typically the first onset dominates perception of location, as per the precedence effect. Young children do show this onset dominance, but if the successive onsets are too far apart in time (e.g., a pronounced echo), they can become confused – sometimes perceiving the later onset as a separate sound or mislocalizing the source. In summary, a singular onset generally yields better localization performance in children, whereas a gradual or complex onset can reduce localization accuracy because the directional cues are less immediate or more ambiguous [18, 19].

These differences raise an important question. Which type of stimulus, noise or words, is better suited for testing directional hearing, especially in children? Given the importance of spatial hearing for children’s safety and communication, it is important to identify the most effective stimuli for assessing sound localization. On one hand, noise stimuli may provide a clearer measure of auditory spatial processing by engaging low-level binaural mechanisms. On the other hand, word stimuli may better reflect real-world listening situations and keep children more engaged during testing. However, few studies have directly compared these stimulus types in pediatric populations, and a clear consensus is lacking [1].

The present study addresses this question by directly comparing speech and noise stimuli in a sound localization task involving children with normal hearing and children with hearing loss using hearing aids. By examining how stimulus type interacts with hearing status and presentation angle, this study seeks to contribute to a better understanding of how directional hearing in children might be assessed in clinical settings.

## Materials and methods

The present study was approved by the local ethics committee of the Medical University of **BLINDED** (no. 1155/2023). Informed written consent was obtained from all participants and their legal guardian before the investigation began.

### Participant cohort

The study included three participant groups. Two groups of children aged between 6 and 10 years, where one group consisted of 31 (15 female) normal hearing children (**Ref**_**6−10**_) with a mean age of 8.5 ± 1.4 years and the second group consisted of 32 (17 female) children with a hearing loss (mean: 8.1 ± 1.2 years) treated with bilateral hearing aids (**HA**_**6−10**_), and a third group consisting of 45 children (27 female) with hearing impairment aged between 11 and 17 years (mean: 13.4 ± 2.3 years), all treated with bilateral hearing aids (**HA**_**11−17**_). The mean age at first hearing aid treatment was 2.2 (SD = 1.5) years in the **HA**_**6−10.**_ group and 2.7(SD = 1.9) years in the **HA**_**11−17**_ group.

**Fig. 1:**
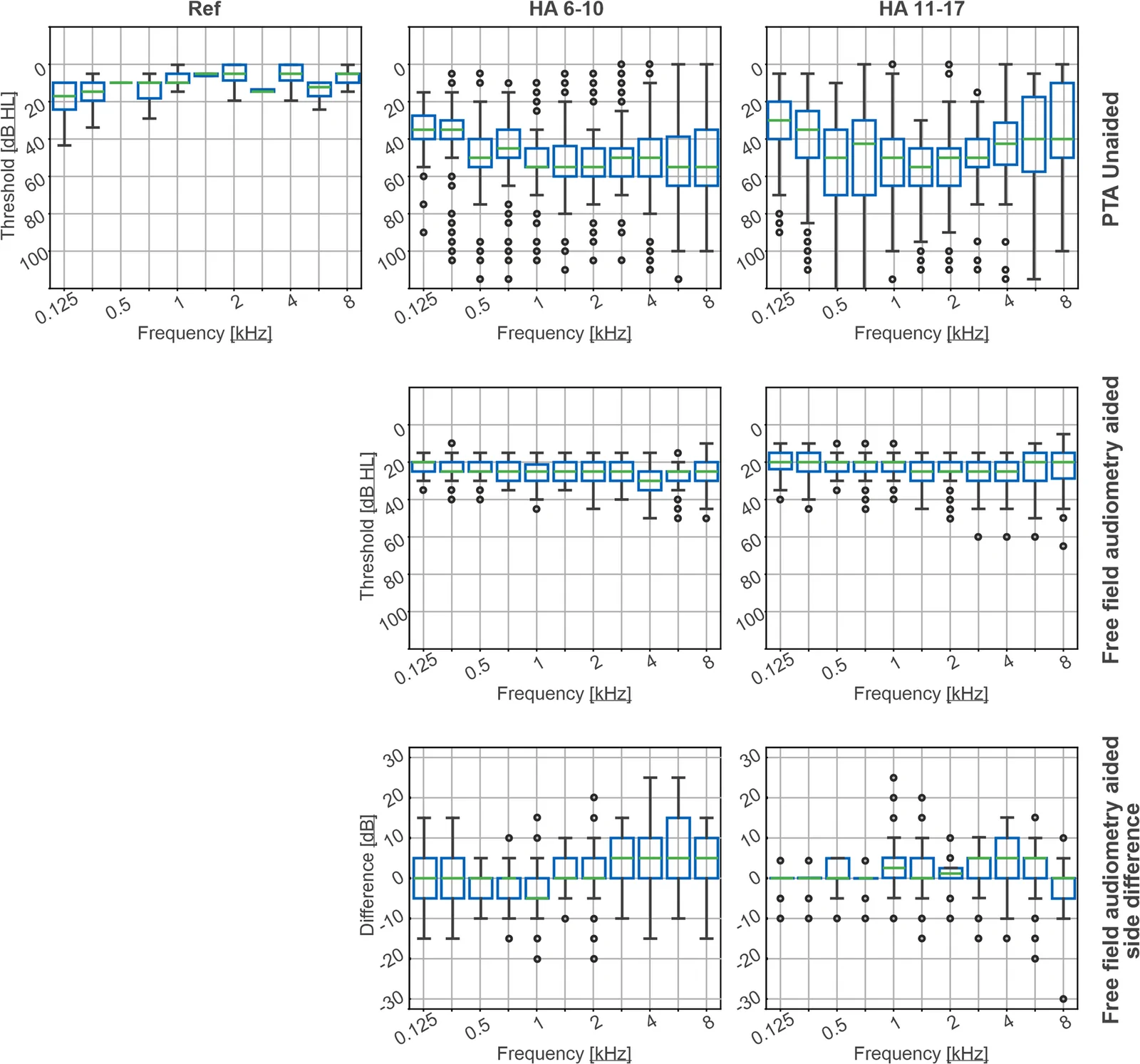
The result of the pure tone audiometric air conduction thresholds was obtained for all three-participant groups. The upper row shows the results of the unaided pure tone audiometry for the reference (first column), HA_6−10_ (middle column), and HA_11−17_ (right column) groups. The second row shows the free field audiometry results in the aided condition. For both rows of plots, data from the left and right ears are pooled. The third row displays the differences in interaural thresholds, calculated as left minus right threshold for each frequency

All the children attend schools, which follow the regular school curriculum. Ear status and hearing was assessed at the outpatient unit of the university hospital **BLINDED** at the Medical University of **BLINDED**. Ear drum impedance measurements and ENT assessments ruled out acute middle ear conditions. The median PTA4 (500–4000 Hz) threshold was 6.3 (± 5.2) dB HL for the normal hearing group, 51.0 (± 19.2) dB HL for the patient group between 6 and 10 years treated with hearing aids, and 52.8 (± 24.4) dB HL for the participants between 11 and 17 years. In best aided condition for patients with hearing aids, the threshold was 28.9 (± 6.5) dB HL and 23 (± 7.2) dB HL for the under-11 and over-10-year groups. The microphone setting for all hearing aids was set to omnidirectional. Audiometric thresholds were assessed for all three participant groups. The results are presented in Fig. 1. The top row displays unaided pure-tone audiometry results for the Reference (left), **HA**_**6−10**_ (center), and **HA**_**11−17**_ (right) groups. The bottom row presents aided free-field audiometry results for the **HA**_**6−10**_ and **HA**_**11−17**_ groups. The third row shows the threshold differences between left and right ears across frequencies.

The results are presented in Fig. 1. The top row displays unaided pure-tone audiometry results for the Reference (left), **HA**_**6−10**_ (center), and **HA**_**11−17**_ (right) groups. The bottom row presents aided free-field audiometry results for the **HA**_**6−10**_ and **HA**_**11−17**_ groups. The third row shows the threshold differences between left and right ears across frequencies. A one-way ANOVA revealed no statistically significant differences between ears in either age group.

### Experimental setup

The sound localization experiment took place in a sound-treated room with five concealed speakers positioned at [−90°, −45°, 0°, 45°, 90°] in the frontal horizontal plane (left to right). Participants responded using a curtain displaying 13 child-friendly symbols (e.g., moon, sun, star), spaced in 15° increments to provide additional response options.

**Fig. 2:**
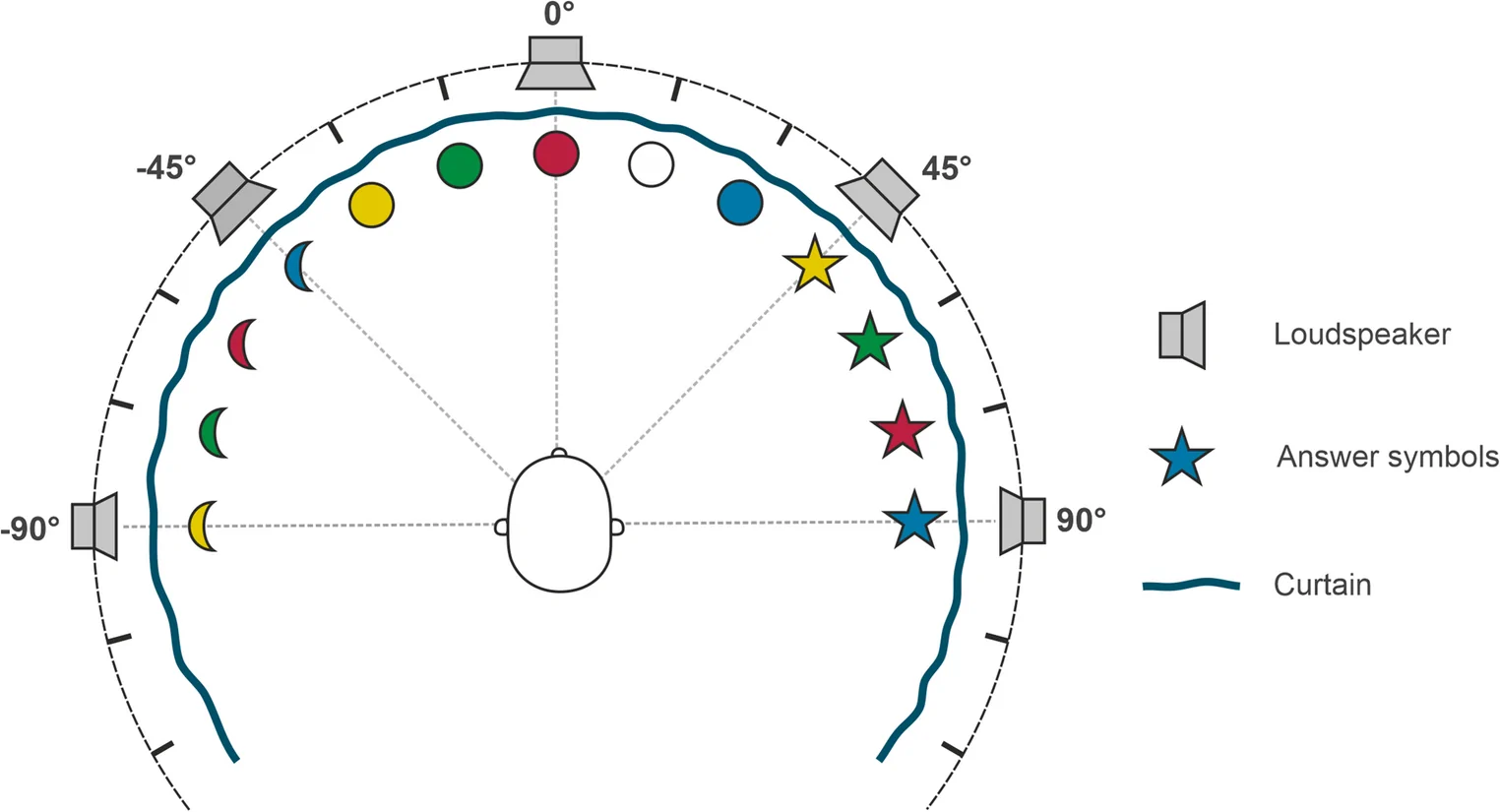
Schematic representation of the experimental setup. The directional hearing test consists of five speakers hidden behind a curtain, which is equipped with 13 child-friendly symbols serving as answer options

To track head position at stimulus onset, participants wore glasses with an integrated front-facing webcam (Tobii Pro Glasses 3). Head positions with a deviation bigger than ± 15° from the front-facing reference position at 0° have been excluded from the analysis. Head positions were manually verified from video recordings by two independent raters, with discrepancies resolved by a third rater. A schematic representation of the setup is presented in Fig. 2.

In addition, to gain an understanding of the preferences of the participants, they have been asked to rate which stimulus group they preferred.

### Stimulus

The broadband noise stimulus used in this study was a 500 ms CCITT noise burst with a 5 ms onset and offset ramp, whereas the speech stimuli consisted of words from the German speech test AAST (Adaptiver Auditiver Sprachtest). For the experiment, the German words “Schneemann” (duration ca. 1.5 s), “Fußball” (duration ca. 1.6 s), “Flugzeug” (duration ca. 1.6 s), and “Eisbär” (duration ca. 1.4 s), have been selected. The stimuli were presented at 60, 65, and 70 dB HL in a randomized order. Each speaker delivered three repetitions per sound level for the noise stimulus, totaling 45 presentations per participant, and three repetitions of each word at each speaker in a randomized order for the speech stimuli, resulting in 65 presentations. The experiments have been performed in the inpatient unit of the University hospital for **BLINDED** at the medical university of **BLINDED** (**BLINDED**). The acoustic stimuli have been presented via a clinical audiometer (Interacoustics AC 40). The Calibration of the AAST speech material was performed according to the instructions provided in the AAST test setup manual. The experiment lasted between 10 and 15 min for each participant, where the word stimuli took up the bulk of the testing time (around 2/3 of the time).

**Fig. 3:**
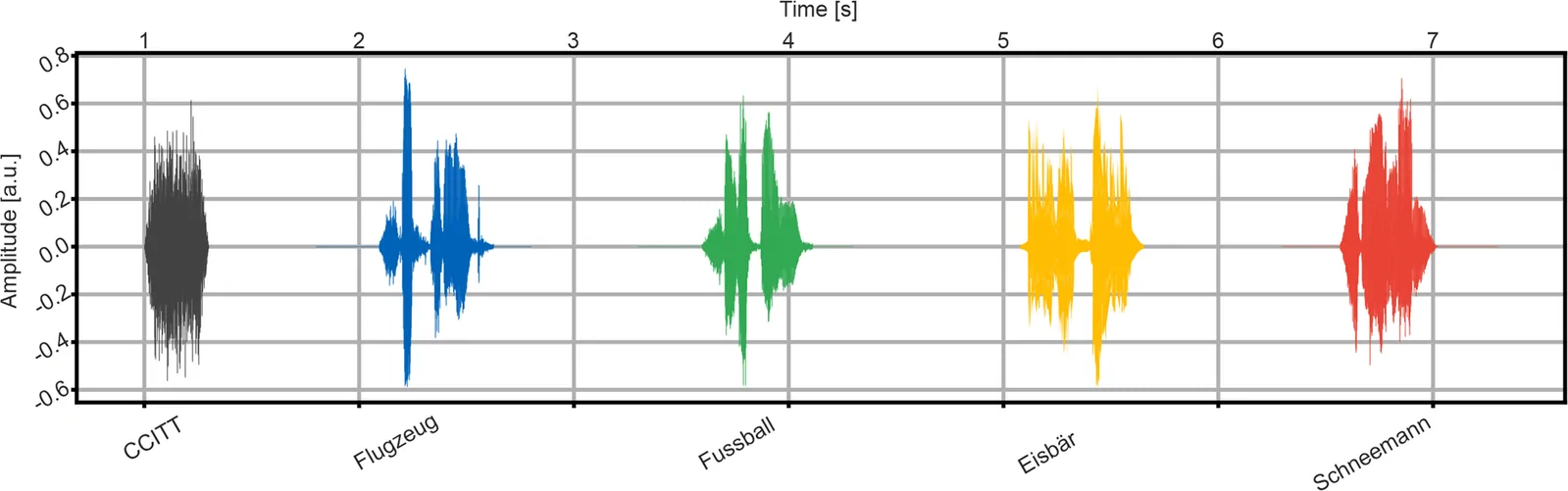
Waveforms of the five auditory stimuli used in the study. The stimuli include one noise sample (“CCITT”, grey) and four spoken German words (“Flugzeug”, “Fussball”, “Eisbär”, and “Schneemann”, colored blue, green, yellow, and red, respectively)

Figure 3 displays the temporal waveform profiles of the five auditory stimuli used in the experiment. The first stimulus (in grey) is a noise signal labeled “CCITT,” which shows a single, continuous sound onset. In contrast, the remaining four stimuli are spoken words, each exhibiting multiple distinct sound onsets that reflect the phonetic structure of the respective word. Each word is shown in a unique color to differentiate them: blue for “Flugzeug,” green for “Fussball,” yellow for “Lenkrad,” and red for “Schneemann.” The x-axis represents time in seconds, while the y-axis shows amplitude in arbitrary units.

### Statistics and data analysis

The root mean square error (RMSE) was calculated for each participant and each presentation angle as the primary dependent variable. RMSE served as a measure of sound localization accuracy, reflecting the deviation between the actual and perceived sound source locations.

Given the repeated-measures nature of the data (multiple angles tested per participant) and violations of normality assumptions as assessed by the Shapiro-Wilk test, linear mixed-effects models (LMMs) were employed instead of traditional ANOVA. LMMs are robust to departures from normality and appropriately account for within-subject correlations in repeated measures designs.

A comprehensive mixed-effects model was first fitted with RMSE as the dependent variable, including Condition (two levels: words, noise) and Angle (five levels: −90°, −45°, 0°, 45°, 90°) as fixed effects, Group (three levels: **Ref**_**6−10**_, **HA**_**6−10**_, **HA**_**7−11**_) as a between-subjects factor, and all two-way and three-way interactions.

To address the research question of whether localization performance differed between stimuli within each group, follow-up mixed-effects models were conducted separately for each group. These within-group models tested the overall effect of Condition (words vs. noise) while accounting for repeated measures across participants.

Subsequently, to examine whether condition effects varied across specific angles, angle-specific mixed-effects models were fitted within each group. For each combination of group and angle, a model was fitted with Condition as the fixed effect. The Benjamini-Hochberg false discovery rate (FDR) correction was applied to control for multiple comparisons across the five angles tested within each group.

All mixed-effects models were implemented using the NumPy (version 1.26.4) and StatsModels package (version 0.14.1) in Python (version 3.12.2). Model convergence was verified for all analyses, and any convergence warnings were noted and addressed.

The root mean square error (RMSE) was calculated for each participant and each presentation angle as the primary dependent variable. RMSE served as a measure of sound localization accuracy, reflecting the deviation between the actual and perceived sound source locations.

A three-way mixed-design analysis of variance (ANOVA) was conducted with Stimulus (two levels: words, noise) and Angle (five levels: [−90°, −45°, 0°, 45°, 90°]) as within-subject factors, and Group (three levels: **Ref**_**6−10**_, **HA**_**6−10**_, **HA**_**11−17**_) as a between-subjects factor. This analysis tested for main effects, all two-way interactions, and the three-way interaction.

Focusing on the research question of whether a difference in localization performance with regard to the stimuli can be observed within the different groups, a follow-up two-way ANOVA was conducted within each group separately, with Stimulus and Angle as within-subject factors. These analyses were designed to investigate how localization performance varied across stimulus types and angles within each age and hearing condition, without reintroducing the Group factor. Where significant Group × Angle interactions were found, additional pairwise comparisons were performed within each group to determine whether RMSE differed significantly across angles for each stimulus condition.

To explore differences between individual word stimuli, pairwise comparisons were also conducted between the four words (Flugzeug, Fussball, Lenkrad, Schneemann) within each group. Appropriate corrections for multiple comparisons (i.e., Benjamini–Hochberg adjusted p-values) were applied across all post hoc tests to control the family-wise error rate.

Assumption checks were conducted prior to all ANOVA analyses. The normality of residuals was assessed using the Shapiro–Wilk test, and homogeneity of variance was evaluated using Levene’s test.

## Results

All assumption checks for normality and homogeneity of variance were violated, as indicated by the Shapiro–Wilk and Levene’s tests. Therefore, linear mixed-effects model tests were used for all post hoc pairwise comparisons to ensure statistical robustness.

**Fig. 4:**
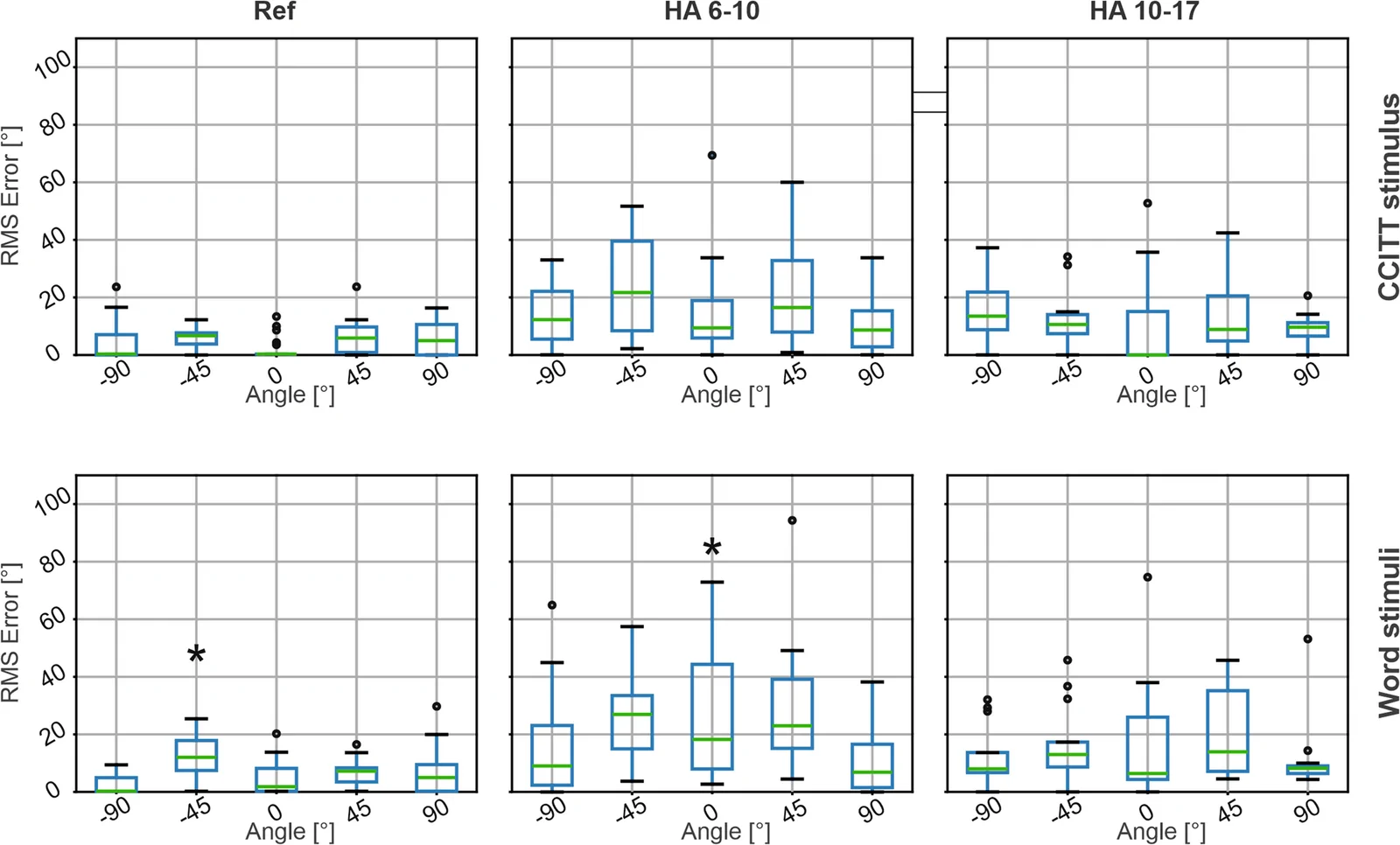
Localization error (RMS) for all test groups. The first row shows the results for the CCITT stimuli, the second row those for the Word stimuli. The first column represents the **REF**_**6–10**_ group, the middle column shows the results for the **HA**_**6–10**_ group, and the third column shows the results for the **HA**_**11–17**_ group. The asterisk indicates angles where the RMS error differs significantly between the word an noise stimulus

Sound localization results for the normal hearing and hearing aid user groups are presented in Fig. 4 (left **REF**_**6−10**_), Fig. 4 (center **HA**_**6−10**_), and Fig. 4 (right **HA**_**11−17**_). Each figure displays localization performance for the noise stimulus (subfigure a, left) and the four word stimuli (subfigure b, right).

**Fig. 5:**
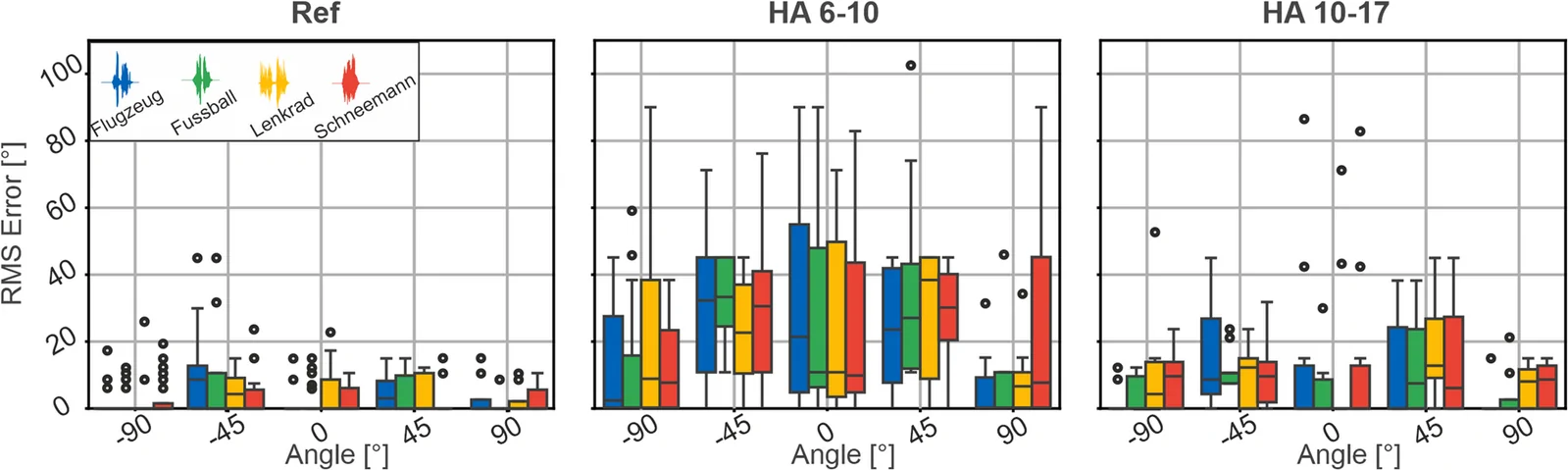
Localization error (RMS) for all test groups across the individual word stimuli. The left panel shows the results for the **REF**_**6–10**_ group, the middle panel for the **HA**_**6–10**_ group, and the right panel for the **HA**_**11–17**_ group

The **REF**_**6−10**_ group exhibited the smallest overall RMS localization error, followed by the **HA**_**11−17**_ group, while the **HA**_**6−10**_ group showed the largest RMS error among all three groups. Visual comparison of the boxplots suggests minimal differences in localization accuracy between stimulus types within each participant group. However, a noticeable exception was observed in the **HA**_**6−10**_ group at 0°, where the RMS error was smaller for the CCITT stimulus compared to the word stimuli.

The results of a linear mixed-effects model analysis revealed a significant main effect of Group, indicating that localization accuracy differed markedly across the three participant groups. Relative to the reference **REF**_**6−10**_ group, both hearing-impaired groups showed substantially higher RMSE values, with larger errors for **REF**_**6−10**_ (β = 15.7, SE = 3.7, z = 4.3, *p* < 0.001) and **HA**_**11−17**_ (β = 11.2, SE = 3.5, z = 3.2, *p* = 0.001). The full results are presented in the Supplementary Table S1.

The main effect of Stimulus was not significant, showing no overall difference in RMSE between words and noise when collapsing across groups (β = 2.14, SE = 1.37, z = 1.57, *p* = 0.116). However, the interaction term for the Group × Stimulus interaction was significant for the **HA**_**6−10**_ group in comparison to the **REF**_**6−10**_ group, demonstrating that the effect of stimulus type differed from that observed in the reference group (β = 4.03, SE = 1.99, z = 2.03, *p* = 0.043). This interaction indicates that the **HA**_**6−10**_ group exhibited a larger increase in localization error for words compared to noise than the reference group. No significant interaction was observed for **HA**_**11−17**_ (β = −1.54, SE = 1.82, z = − 0.85, *p* = 0.397).

The random-effects term confirmed considerable variability between participants, consistent with individual differences in localization performance (variance = 69.81).

In order to estimate the effect of the stimulus a linear-mixed-model analysis was performed comparing the stimuli within all three test-groups. The analysis showed a significant effect for the **REF**_**6−10**_ group (β = 2.14, SE = 0.69, z = 3.08, *p* = 0.002) and for the **HA**_**6−10**_ group (β = 6.17, SE = 2.08, z = 2.96, *p* = 0.003). No significant effect was found in the **HA**_**11−17**_ group (β = 0.60, SE = 1.09, z = 0.55, *p* = 0.58). The full results are presented in Supplementary Table S2.

A parametric t-test was used to calculate the pairwise comparison between each angle and stimulus within the three groups. The pairwise comparisons revealed statistically significant differences in the RMS Error for the comparison between word and noise stimuli in the normal hearing reference group at the presentation angles − 45° with *p* < 0.001. The mean RMSE was lower for the CCITT stimulus in both cases (5.13° vs. 11.8°). Additionally, a statistically significant difference was found at 0° for the **HA**_**6−10**_ group at *p* = 0.03, where the mean RMSE was again lower for the CCITT noise stimulus (20.1° vs. 31.5°). No other angle-wise differences between stimulus types reached significance. The full results of the pairwise comparisons are presented in Supplementary Table S3.

A comparison of the RMS error across individual word stimuli is shown in Fig. 5. The results indicate no observable differences in localization performance between the four words across all presentation angles and groups.

One-way ANOVA comparisons between the four word stimuli within each group (as shown in Fig. 5) did not reveal any significant differences in localization performance across angles and test words. The results of the statistical analysis are show in the Supplementary Table S4.

## Discussion

The present study set out to explore whether noise or word stimuli are more suitable for assessing sound localization abilities in children, particularly in relation to hearing status. This question holds clinical relevance, as both types of stimuli are used in localization testing, yet their comparative suitability remains underexplored in pediatric populations.

The results of the linear mixed-effects model revealed no significant main effect of stimulus type, indicating that localization performance did not differ between word and noise stimuli across the three groups.

In contrast, the model showed a significant main effect in the comparison between the three test groups, demonstrating that localization performance differed between the three participant groups. The interaction between stimulus type and patient group was significant for the **HA**_**6–10**_ group, indicating that the effect of stimulus type in this group differed from that of the reference group (**Ref**_**6–10**_). No such interaction was observed for the **HA**_**11–17**_ group, suggesting that its stimulus-related performance pattern did not differ significantly from the reference group.

These results are accompanied thee group-specific effects, pointing to potential differences of one stimulus type over the other in specific populations. The follow up linear mixed-effects model analysis of the difference in localization performance within the groups with regard to the stimulus type revealed a significant effect for the **Ref**_**6–10**_ and **HA**_**6–10**_ group.

The analysis revealed no significant differences in the teenage (aged 11 to 17 years) hearing aid group (**HA**_**11–17**_). The present study is consistent with maturation, by finding a lower RMSE for the **HA**_**11–17**_ group compared to the **HA**_**6–10**_ group, the maturation of the localization abilities with increasing age [3].

Pairwise comparisons of the localization abilities at each speaker position, within the three test groups, found no significant differences in the localization abilities between noise or word stimuli in the **HA**_**11–17**_ group of children with hearing aids.

However, although we did not find any significant differences between the stimuli on a group level, the analysis found a significant difference between word and noise stimuli at 0° in the **HA**_**6–10**_ group, showing a worse localization performance for the word stimuli. In light of the marginal significance and the absence of an established mechanism, we are unable to provide a meaningful interpretation of this finding.

A futher notable finding of our study was the presence of an asymmetry in sound localization performance within the reference group (**Ref**_**6–10**_), with a significant difference between word and noise stimuli and a lower RMSE for the noise stimuli presented at −45°. A possible explanation for this result may lie in the brain’s lateralized processing of auditory information. Spierer et al. [20] found that patients with right hemisphere brain damage exhibited greater difficulties in sound localization compared to those with left-sided or no brain damage, highlighting the right hemisphere’s dominant role in spatial hearing. Spierer et al. [20] emphasized the importance of the right auditory cortex in the processing of interaural time differences (ITDs), which are crucial cues for localization.

However, ITD cues are generally poorly transmitted through hearing aids, which may explain why this asymmetric difference in the localization abilities between noise and word stimuli was only observed in the normal-hearing reference group [21]. Furthermore, several studies have shown that brain maturation occurs at different rates across hemispheres, with the right hemisphere in general showing faster development [22, 23], potentially contributing to lateralization effects.

The commonly observed right ear advantage for speech perception (e.g., Moncrieff et al., 2011 [24]; Pittman et al., 2024 [25]) suggests that speech is predominantly processed in the left auditory cortex, whereas non-speech sounds are more strongly associated with the right auditory cortex. Taken together, the effects of brain maturation, hemispheric specialization for speech processing, and the role of ITDs may explain the higher localization performance for noise stimuli in the normal-hearing reference group.

The comparison of the localization performance of the four different words did not reveal any significant differences. This contradicts the idea that signals with more sound onsets, such as words, provide better localization potential than a single onset noise stimulus.

Overall, our findings suggest that neither noise nor word stimuli are universally better suited for assessing localization accuracy in children.

Instead, the choice of stimulus may depend on developmental factors and hearing status. Noise stimuli may offer a more undisrupted measure of binaural hearing mechanisms and a shorter test time, especially in younger or hearing-impaired children, while word stimuli may provide a more real-world scenario to test spatial hearing, particularly relevant for school-age children who rely heavily on speech in their daily environments.

From a clinical perspective, incorporating both noise and word-based localization tasks may yield a more comprehensive assessment of spatial hearing abilities. Using multiple stimulus types allows clinicians to differentiate between perceptual limitations and cognitive or linguistic challenges, which may be especially valuable in hearing-impaired pediatric populations.

A limitation of this study is the absence of an age-matched normal-hearing control group for the 11–17-year cohort, limiting the ability to disentangle age-related maturation effects from group differences. However, the findings observed in the younger control group may generalize to older age groups.

## Conclusion

This study demonstrates that both noise and word stimuli are valid for assessing sound localization in children, with each offering distinct advantages depending on age and hearing status. Using a combination of stimulus types may provide the most accurate and informative evaluation of spatial hearing in pediatric clinical settings.

## Supplementary Information

Below is the link to the electronic supplementary material.

Supplementary Material 1 (DOCX 29.6 KB)
