# Supplementary material for: Speech versus noise in pediatric sound localization
Source: Eur Arch Otorhinolaryngol. 2026 Jul 19;283(9):6037–45. doi: 10.1007/s00405-026-10415-5 (PMC13615084; doi:10.1007/s00405-026-10415-5)
Supplement: Supplementary file 1 [file 405_2026_10415_MOESM1_ESM.docx]

Supplementary Data:
Table S1. Results of the linear mixed-effects model examining the effects of Group and Stimulus on localization accuracy (RMSE). The model included fixed effects of Group, Stimulus, and their interaction. Coefficients represent differences relative to the reference levels (Group: REF6-10  Stimulus: Noise). Significant p-values are highlighted in bold.
Effect
Coef.
Std. Err.
p
Stimulus
2.14
1.4
0.11
Group HA6-10
15.7
3.7
< 0.001
Group HA11-17
11.2
3.5
0.001
Stimulus x Group HA6-10
4.0
2.0
0.04
Stimulus x Group HA11-17
-1.5
1.8
0.4


Table S2. Results of the linear mixed-effects model examining the effect of the acoustic Stimulus on localization accuracy within the participant groups. Coefficients represent differences relative to the reference level (Noise). Significant p-values are highlighted in bold.
Group
Coef.
Std. Err
p
REF6-10  
2.1
0.7
0.002
HA6-10
6.2
2.1
0.003
HA11-17
0.6
1.1
0.58

Table S3 P-values of the pairwise comparison of the stimulus type for fixed angles for all three test groups.
Group
-90°
-45°
0°
45°
90°
Ref6-10
0.56
<0.001
0.09
0.09
0.89


HA6-10
0.85
0.07
0.03
0.17
0.18


HA11-17
0.86
0.86
0.74
0.06
0.11

Table S4. Results of the one way Anova with the factor word (“Schneemann”, “Fußball”, “Flugzeug”, and “Eisbär”) for all groups.
Group

-90°
-45°
0°
45°
90°
Ref6-10
F(3,107)
0.72
1.14
0.93
0.02
0.72

p
0.54
0.33
0.43
0.99
0.54
HA6-10
F(3,132
0.48
0.64
0.34
0.40
0.93

p
0.69
0.58
0.79
0.75
0.42
HA11-17
F(3,216)
0.08
0.39
0.19
0.78
0.80

p
0.97
0.75
0.90
0.51
0.49
